# Hardy-Rand-Rittler colour vision testing in cone and cone-rod dystrophies: correlation with structural and functional outcome measures

**DOI:** 10.1038/s41433-024-03584-2

**Published:** 2025-01-16

**Authors:** Brindhan Tharmarajah, Elisa E. Cornish, Jonathan Nguyen, Elizabeth Barnes, Kate E. Leahy, Anagha Vaze, Robyn V. Jamieson, John R. Grigg

**Affiliations:** 1 Save Sight Institute, Faculty of Medicine and Health, The University of Sydney, Sydney, NSW Australia; 2 Sydney Eye Hospital, Sydney, NSW Australia; 3 Eye Genetics Research Unit, Children’s Medical Research Institute, The Children’s Hospital at Westmead, Sydney, NSW Australia; 4 NHMRC Clinical Trials Centre, University of Sydney, Sydney, NSW Australia; 5 Disciplines of Genomic Medicine & Child and Adolescent Health, The University of Sydney, Sydney, NSW Australia

**Keywords:** Eye manifestations, Predictive markers, Retinal diseases

## Abstract

**Purpose:**

To determine how Hardy-Rand-Rittler (HRR) colour vision testing correlates with visual functional and structural assessments in Cone and Cone-Rod Dystrophy.

**Methods:**

Thirty-four Cone and 69 Cone-Rod Dystrophy patients diagnosed by electroretinography (ERG) at the Save Sight Institute in Sydney were included in a retrospective analysis. Each patient’s HRR colour vision test scores were compared with markers of cone and rod system function including visual acuity (VA), ERG responses, changes on Spectral Domain Optical Coherence Tomography (OCT) and Fundus Autofluorescence.

**Results:**

The number of plates identified on HRR testing correlated with logMAR best-corrected distance VA; r(101) = −0.49, *p* < 0.0001. HRR scores correlated with markers of cone and macula function including OCT Ellipsoid Zone Gap Width, Central Macular and Outer Nuclear Layer Thickness, Full Field ERG 30 Hz flicker amplitudes, light adapted 3.0 b-wave amplitudes and Pattern ERG 15- and 30-degree p50 amplitudes.

**Conclusion:**

HRR colour vision testing correlates with structural and functional measures in Cone and Cone-Rod Dystrophy. HRR colour vision testing provides a simple clinic-based option to monitor disease changes in Cone and Cone-Rod Dystrophy patients, especially when ERG testing is not available.

## Introduction

One subgroup of inherited retinal diseases is progressive Cone Dystrophy (CD) and Cone-Rod Dystrophy (CRD). CD is marked by initial loss of cone function with potential later rod system involvement. In contrast, CRD involves loss of both cone and rod function from the outset with cone dysfunction being more prominent at presentation [1–3]. Patients with CD and CRD (CORD) present with reduced vision, photophobia and colour vision loss in adolescence [4, 5]. Most CORD patients become legally blind before age 48 [6]. Current markers of disease progression include visual acuity (VA) and changes on Electroretinography (ERG), Fundus Autofluorescence (FAF) and Spectral Domain Optical Coherence Tomography (OCT) [7]. However, ERG is not commonplace in ophthalmic practices. Finding another easily accessible test to monitor disease progression in CORD patients is desirable. Hardy-Rand-Rittler (HRR) is a commonly available colour vision test that has been found useful in the early detection of CD and CRD [5, 8].

No studies have investigated the ability of colour vision tests to monitor disease progression in CORD. This study will determine if there is a correlation between the severity of colour vision loss with HRR testing and markers of cone and rod dysfunction in patients with CORD. These include VA and changes on ERG, FAF and OCT. This will provide an understanding of how colour vision deteriorates in this cohort. It will also determine whether HRR can be used to monitor disease progression. As this bedside test is non-invasive, low-cost and widely available, it provides a simple option to monitor disease progression especially in practices which lack ERG.

## Methods

A retrospective analysis of patients with CD and CRD who attended the Save Sight Institute, The University of Sydney, was completed. The diagnosis of CD was made if they had a progressive decline in VA with reduced cone responses with relative preservation of rod responses on Full Field ERG compared to reference values. The diagnosis of CRD was made if they had reduced cone and rod responses on Full Field ERG with cone function equally or more severely affected than rod function [9]. Patients were included if they had completed the Richmond HRR Fourth Edition colour vision test at their most recent visit. The study was conducted in compliance with the Declaration of Helsinki. Ethics approval was obtained from the South-East Sydney Local Health District Human Research Ethics Committee.

The HRR test was performed monocularly under daylight illumination with appropriate refractive correction. Patients were required to identify the demonstration plates before proceeding. Diagnostic plates 11−24 were then completed and the total number of correct figures scored out of 28. Subjects were given two seconds to respond to each HRR plate. If patients could not see any plates, they were given a score of 0. HRR was classified as normal if no symbols were missed. Colour defects were graded as mild (1 or more errors in plates 11–15); moderate (1 or more errors in plates 16–18 for Protan/Deutan [P/D] defects, or in 21–22 for tritan defects) or severe (1 or more errors in plates 19–20 for P/D defects, or in 23–24 for tritan defects) as per HRR instructions (Supplementary Fig. 1) [5].

Each patient’s colour vision testing scores were compared with markers of retinal function from their right eye at the same clinical examination. This included best-corrected Maclure near and logMAR-converted Snellen best-corrected distance VA, Pattern ERG 15- and 30-degree p50 amplitudes as well as Full Field ERG LA 30 Hz, DA 0.01, DA 3.0 and LA 3.0 a and b wave amplitudes and latencies [6,10–12]. ERG was performed in accordance with the International Society for Clinical Electrophysiology of Vision Standards with gold foil active electrodes and a Diagnosys Colour-Dome system running Espion E3 software version 6.64.15 (Diagnosys LCC, Lowell, MA) [11]. The ERG data of 10 patients who were tested with skin electrodes was excluded. If VA was less than 6/60, the patient’s ability to count fingers, see hand movements or light was recorded and converted to LogMAR values using the methodology of Day et al. [13]. Patients were not excluded based on low VA as the cutoff values for HRR colour vision testing are variable in the literature [14].

HRR scores were also compared with OCT and FAF markers of declining cone and rod function from the same visit. Changes on Spectralis Spectral Domain OCT (Heidelberg Engineering, Heidelberg, Germany) scans of the macula including decreasing Central Macular Thickness (CMT) and Outer Nuclear Layer (ONL) thickness and increasing Ellipsoid Zone (EZ) gap width were measured [6, 10,15–17]. CMT was defined as the distance between RPE-Bruch’s Membrane complex and the internal limiting membrane at the fovea and measured using the ‘Centre’ value on the Early Treatment Diabetic Retinopathy Study map created by the inbuilt OCT software [17]. CMT was used as a key marker of declining cone function given its significant correlation with VA and ERG photopic responses [7, 18]. EZ gap was defined as the longest continuous horizontal disruption in the EZ band including residual EZ if a gap was seen either side and was used as a proxy measure for foveal cone photoreceptor loss [17]. It was measured from the first interruption point nasal to the foveal centre to the last interruption point temporal to the foveal centre using the OCT software’s embedded digital callipers and 1:1 μm setting. In patients who underwent FAF retinal imaging with an Optos ultrawide field imaging device (Optos Inc, Dunfermline, UK), the diameter and area of abnormal macular FAF (outer hyper-autofluorescence and inner hypo-autofluorescence) was measured manually using Optos software without blinding. FAF changes were measured as they are known to correlate with declining rod function and visual field loss in CORD [7, 9].

Prism statistics software, version 10.0.2 (GraphPad Software Inc, Boston MA, USA) was used to identify correlations between HRR scores and the listed clinical variables. Given the non-Gaussian distribution of the data, non-parametric Spearman’s Rank Correlation Coefficient, Mann–Whitney *U*, Kruskal–Wallis and Dunn’s tests were used with a two-tailed *p* value and statistical significance set at *p* ≤ 0.05. A secondary analysis was completed with Bonferroni correction for comparison of 19 variables (adjusted *p* ≤ 0.0026).

## Results

### Clinical characteristics of study population

Thirty-four CD and 69 CRD patients were included in the study. CD patients age ranged from 6–68 years with an average of 30 years. CRD patients age ranged from 6–82 years with an average of 41 years. 58.8% of CD and 57.9% of CRD patients were male. LogMAR-converted best-corrected Snellen distance VA of CD patients ranged from −0.2 to 1.3 with a mean acuity of 0.59. VA of CRD patients ranged from −0.2 to light perception with a mean acuity of 0.77. 17.6% of CD patients and 33.3% of CRD patients had a logMAR VA greater than or equal to 1.

One hundred and three CORD patients completed HRR testing. Average HRR total, PD and tritan section scores were 7.2/28, 4.6/20 and 2.5/8, respectively. 6.8% of patients had a normal P/D grade while 12.6% had a mild P/D defect, 5.8% a moderate defect and 74.8% a severe defect. 25% of patients had a normal tritan grade while 5% and 70% had a moderate and severe defect, respectively.

In the CD group, average HRR total, PD and tritan section scores were 10/28, 6.5/20 and 3.6/8 respectively. 8.8% of patients had a normal P/D grade while 17.7% had a mild P/D defect, 8.8% a moderate defect and 64.7% a severe defect. 35.4% of patients had a normal tritan grade while 5.8% and 58.8% had a moderate and severe defect respectively.

In the CRD group, average HRR total, PD and tritan section scores were 5.8/28, 3.8/20 and 2/8 respectively. 5.9% of patients had a normal P/D grade while 10.1% had a mild P/D defect, 4.3% a moderate defect and 79.7% a severe defect. 20.3% of patients had a normal tritan grade while 4.3% and 75.4% had a moderate and severe defect respectively.

### Comparison of HRR test results with markers of cone and rod function

**Fig. 1:**
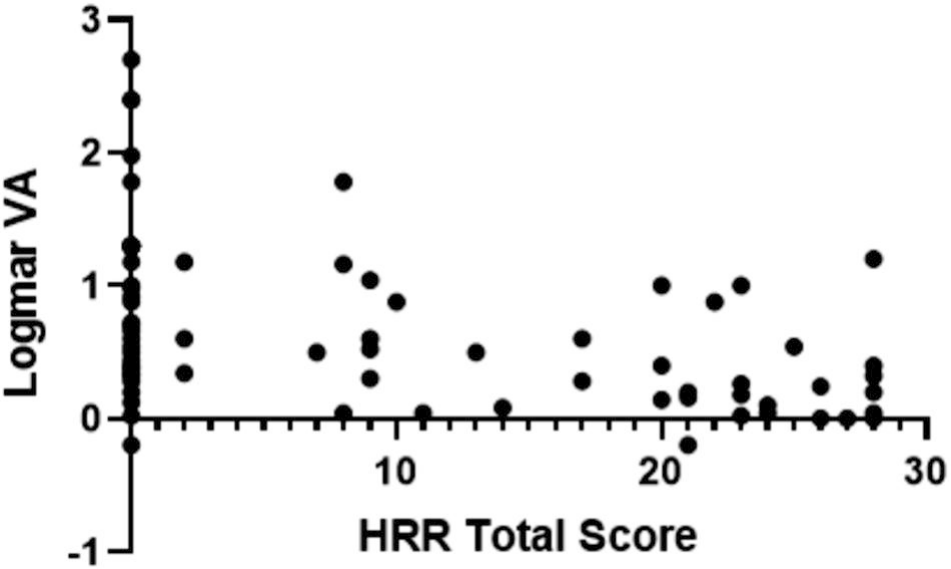
Association between Hardy-Rand-Rittler total score and distance visual acuity In The CORD group. *VA* best-corrected distance visual acuity; *Logmar* Logarithm of the minimum angle of resolution; *HRR* Hardy-Rand-Rittler.

**Fig. 2:**
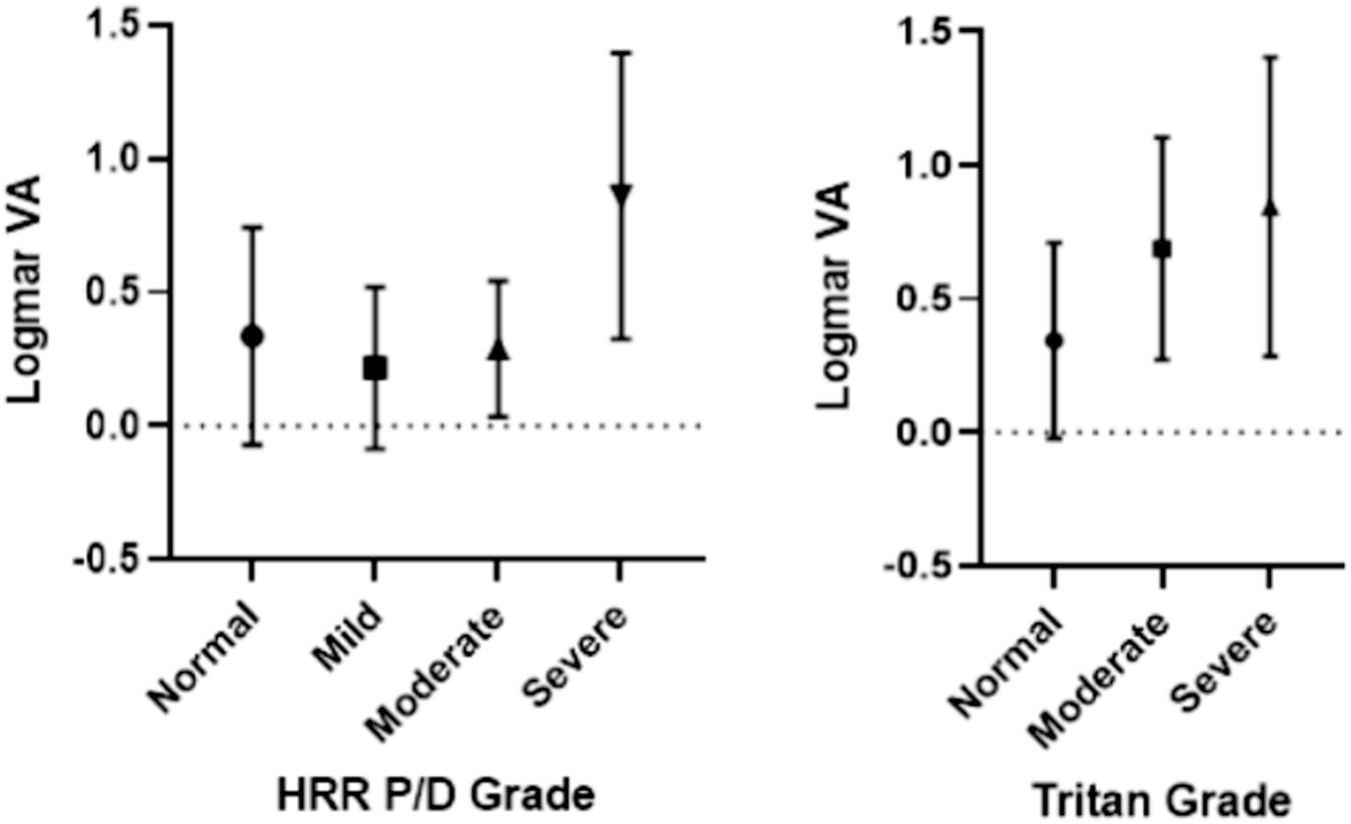
Association between Hardy-Rand-Rittler Protan/Deutan and tritan grades and distance visual acuity In the CORD group. *VA* Best-Corrected Distance Visual Acuity; *Logmar* Logarithm of the minimum angle of resolution; *HRR* Hardy-Rand-Rittler; P/D Protan-Deutan. Means and 1 standard deviation are shown.

Significant correlations were found between HRR results and markers of retinal function (Table 1) including VA in the CD, CRD and combined CORD group (Figs. 1–2).

**Table 1:**
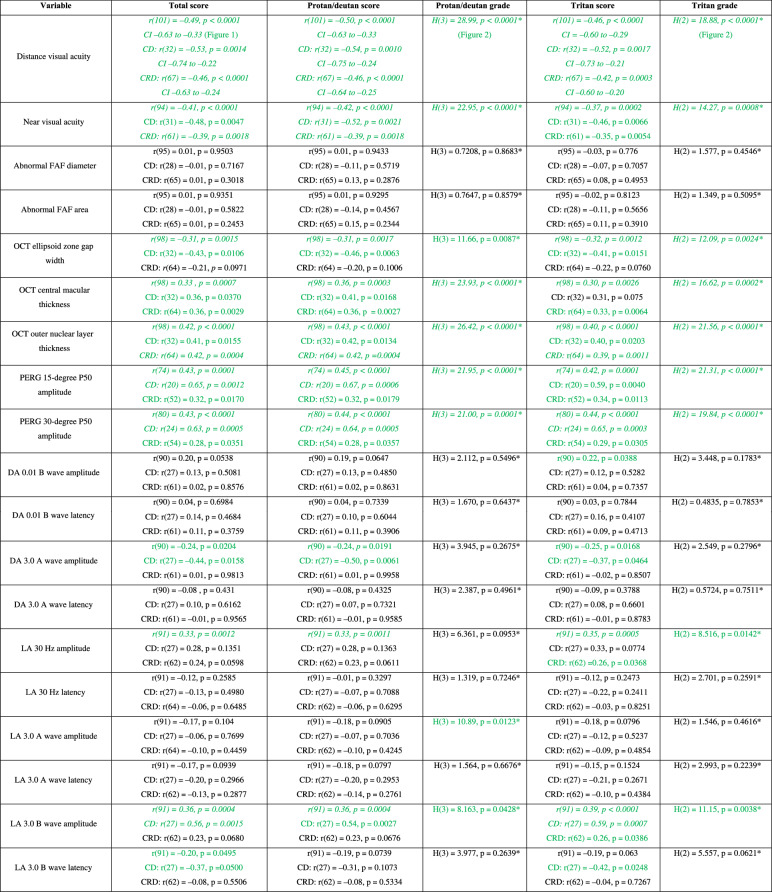
Comparison of Hardy-Rand Rittler results with markers of cone and rod function In The CORD group.

Spearman’s Rank Correlation Coefficient was used for all comparisons except for those marked with an * which indicates the use of Kruskal–Wallis and Dunn’s tests. Green text indicates a significant correlation (*p* ≤ 0.05) while italicised text indicates a significant correlation with Bonferroni correction (adjusted *p* ≤ 0.0026).

*FAF* fundus autofluorescence, *OCT* Optical Coherence Tomography, *PERG* pattern electroretinography, *DA* dark adapted, *LA* light adapted, *CD* cone dystrophy, *CRD* cone-rod dystrophy, *CI* confidence interval.

## Discussion

HRR testing revealed colour vision disturbance in CORD patients. Only 6.8% and 25.2% of patients who completed HRR testing had a normal P/D and tritan grade, respectively, highlighting a greater disturbance of the P/D axis. This matches the findings of Thiadens et al. and highlights the heterogenous impairment of all three colour vision axes in patients with CORD [5]. CRD patients had more severe visual dysfunction than CD patients with a mean VA of 0.88 versus 0.59. They also had more severe color vision dysfunction, with 79.7% and 75.4% of CRD patients having severe P/D and tritan defects, versus 64.7% and 58.8% of the CD group, respectively. This is in keeping with previous studies showing that CRD patients have more severe disease on psychophysical testing and earlier onset of legal blindness at around 23 years of age compared with 48 years for CD patients [4, 6]. HRR and Lanthony Panel D-15 testing of 181 CORD patients by Thiadens et al. identified a progressive decline in colour vision. 91% of CD and 95% of CRD patients developed severe colour defects 10 years after diagnosis [19]. It is therefore important to monitor all 3 colour vision axes.

There is uncertainty regarding the ability of colour vision testing to monitor disease progression in CORD. A study of 37 CD patients found that HRR, Ishihara and the Lanthony Desaturated Panel D-15 test had high sensitivity, specificity, positive predictive value and discriminative accuracy in identifying CD patients but that there was no significant correlation between colour defect severity in either test and ERG cone responses, VA or visual field defects [5]. Only one study found that HRR could quantify cone dysfunction with CD progression. However, this study was not published in full, online and did not state which markers of disease progression HRR was compared with to reach this conclusion [8].

In our study, HRR testing was found to correlate with VA and markers of cone and macula function in CORD patients. There was a significant correlation between HRR total score, P/D score and grade, tritan score and grade, and distance VA, near VA, EZ gap width, CMT, ONL thickness, PERG 15- and 30-degree p50 amplitude and LA 3.0 b wave amplitude. HRR total score, P/D score and tritan score and grade also correlated with LA 30 Hz amplitude, a key marker of disease onset [6, 10]. Hence, unlike Thiadens et al., a significant correlation was found between HRR defect severity and VA and ERG cone and macula responses, making it a useful functional marker in CORD [5]. No correlation was found between HRR total score, P/D or tritan score and markers of rod function including abnormal FAF diameter or area, DA 0.01 b wave amplitude and latency and DA 3.0 a wave latency. HRR can therefore be used as a marker of cone and macula function in CORD patients, especially when ERG is not available [7, 11].

A sub-analysis of the CRD group showed reduced correlation of HRR scores with markers of retinal function. For example, there was no significant correlation with OCT EZ gap width, DA 3.0 a wave amplitude, LA 3.0 b wave amplitude and latency. The CD group had no significant correlation between OCT CMT and HRR tritan score. Both subgroups had no significant correlation with LA 30 Hz amplitude, likely reflecting their small sample sizes. However, both groups maintained significant relationships between HRR scores and other variables including VA, near VA and pattern ERG responses, indicating a correlation with macula and cone function [10, 11]. This is the first study to show that HRR can be used as a marker of retinal function in both CD and CRD patients.

The use of a Bonferroni correction in this study created differing results. Given the study’s small CD sample size of 34 patients and the risk of creating false negatives with Bonferroni correction, the results of analysis without adjustment are shown above. When a Bonferroni correction was applied (adjusted *p* ≤ 0.0026) to reduce risk of false positives in the context of multiple comparisons, HRR maintained a significant correlation with VA, CMT and cone and macula ERG changes in the CORD group [20]. However, CD and CRD subgroups showed reduced correlation between HRR scores and markers of retinal function, likely due to their smaller sample sizes. CD and CRD lie on a spectrum of disease and both the CD and CRD subgroups maintained a significant relationship between their HRR scores and markers of retinal function including VA with Bonferroni correction [4, 10]. As such, HRR can still be confidently used to monitor disease changes in CD and CRD patients.

Figures 1 and 2 highlight how greater colour vision loss on HRR testing is associated with reduced VA. This reflects the critical nature of foveal cone photoreceptors for colour vision and VA [21]. This also indicates that impaired VA could cause colour vision abnormalities in CORD patients. However, studies have shown that patients can have normal VA and poor colour vision and vice versa [12, 14]. Furthermore, Foote et al. found that VA and foveal cone spacing were weakly correlated until cone density was reduced to 40% below normal in patients with inherited retinal disease. This suggests that VA is not a sensitive measure of early foveal cone photoreceptor loss [22]. Hence, colour vision should be monitored as an individual marker of disease change in CD and CRD patients. Further studies are needed to determine if colour vision changes precede a decline in other functional outcomes.

Limitations of our study include the small sample size which reduced the statistical power of CD and CRD subgroup analyses. As many patients did not return for follow-up, we were unable to compare colour vision test scores over time, limiting our analysis of progression. As the screening plates were not included in the HRR assessment, mild tritan defects were not included in the analysis, limiting tritan assessment.

Our study did not review other colour vision tests such as the Ishihara pseudoisochromatic test, the Farnsworth-Munsell 100 Hue Test or the Nagel Anomaloscope. While the Ishihara test is commonly available, it cannot assess the tritan colour vision axis or quantify defect severity. While the Farnsworth–Munsell 100 Hue test and the Nagel Anomaloscope are more capable of diagnosing and monitoring the severity of acquired and congenital colour vision defects, these tests were not included given their time-consuming nature and lack of availability in most practices [5, 23, 24]. We chose to analyse the HRR test as Thiadens et al. found it to have the highest discriminative accuracy in detecting CORD patients; even in patients with low vision, when compared to the Ishihara, Lanthony Desaturated D15 and Farnsworth Munsell D15 colour vision tests [5]. The HRR test also has the inherent advantages of being available in most ophthalmic practices, being able to detect and quantify colour defects in all three colour vision axes, and be used in children and illiterate adults [5, 23, 24].

In summary, colour vision is affected in all three axes in CORD. A significant correlation was found between the severity of colour vision loss on HRR testing and functional and structural markers of CORD including distance and near VA, OCT EZ gap width, CMT and ONL thickness, Full Field ERG 30 Hz flicker amplitudes, light adapted 3.0 b wave amplitudes and Pattern ERG responses. This indicates that cone and macula function in CORD patients can be monitored through HRR colour vision assessment, especially in practices where ERG testing is not available. HRR is a bedside colour vision test that provides a quick, non-invasive, low-cost, widely available option to monitor all 3 colour vision axes in CORD patients alongside standard investigations including ERG. In low resource settings where ERG testing is not available, HRR colour vision testing provides practitioners with another tool to better monitor disease progression in this cohort.

## Summary

### What was known before

- Colour vision is known to deteriorate in patients with Cone and Cone-Rod Dystrophy
- No studies have investigated the ability of colour vision testing to monitor disease progression in Cone and Cone-Rod Dystrophy.

### What this study adds:

- Colour vision is affected in all three axes in Cone and Cone-Rod Dystrophy.
- Hardy-Rand-Rittler colour vision testing correlates with structural and functional measures in Cone and Cone-Rod Dystrophy.
- Hardy-Rand-Rittler colour vision testing provides a simple, clinic-based option to monitor disease changes in patients with Cone and Cone-Rod Dystrophy, especially when electroretinography testing is not available.

## Supplementary information

Supplementary Figure 1 - Hardy-Rand-Rittler Scoring Sheet

## References

[1] Roosing S, Thiadens AA, Hoyng CB, Klaver CC, den Hollander AI, Cremers FP. Causes and consequences of inherited cone disorders. Prog Retin Eye Res. 2014;42:1–26. 10.1016/j.preteyeres.2014.05.001. 24857951 10.1016/j.preteyeres.2014.05.001

[2] Simunovic MP, Moore AT. The cone dystrophies. Eye. 1998;12:553–65. 10.1038/eye.1998.145. 9775217 10.1038/eye.1998.145

[3] Hamel CP. Cone rod dystrophies. Orphanet J Rare Dis. 2007;2:7. 17270046 10.1186/1750-1172-2-7 PMC1808442

[4] Nash BM, Wright DC, Grigg JR, Bennetts B, Jamieson RV. Retinal dystrophies, genomic applications in diagnosis and prospects for therapy. Transl Pediatr. 2015;4:139. 26835369 10.3978/j.issn.2224-4336.2015.04.03 PMC4729094

[5] Thiadens AA, Hoyng CB, Polling JR, Bernaerts-Biskop R, van den Born LI, Klaver CC. Accuracy of four commonly used colour vision tests in the identification of cone disorders. Ophthalmic Epidemiol. 2013;20:114–22. 23510316 10.3109/09286586.2012.759596

[6] Gill JS, Georgiou M, Kalitzeos A, Moore AT, Michaelides M. Progressive cone and cone-rod dystrophies: clinical features, molecular genetics and prospects for therapy. Br J Ophthalmol. 2019;103:711–20. 30679166 10.1136/bjophthalmol-2018-313278 PMC6709772

[7] Kanda S, Hara T, Fujino R, Azuma K, Soga H, Asaoka R, et al. Correlation between fundus autofluorescence and visual function in patients with cone-rod dystrophy. Sci Rep. 2021;11:1–7. 33479408 10.1038/s41598-021-81597-7 PMC7820325

[8] Thiadens AA, Van Lith-Verhoeven J, Bernaerts R, Polling J, Simonsz H, Klaver C. Which colour vision test should be used in progressive cone dystrophy. Abstr ARVO Annu Meet. 2007;48:13.

[9] Oishi M, Oishi A, Ogino K, Makiyama Y, Gotoh N, Kurimoto M, et al. Wide-field fundus autofluorescence abnormalities and visual function in patients with cone and cone-rod dystrophies. Investig Ophthalmol Vis Sci 2014;55:3572–7. 24845635 10.1167/iovs.14-13912

[10] Puech B, De Laey J, Holder GE. Inherited chorioretinal dystrophies: a textbook and atlas. Chapter 2, p13–18 (Springer, 2014).

[11] Robson AG, Nilsson J, Li S, Jalali S, Fulton AB, Tormene AP, et al. ISCEV guide to visual electrodiagnostic procedures. Doc Ophthalmol 2018;136:1–26. 29397523 10.1007/s10633-017-9621-y PMC5811581

[12] Sadowski B, Zrenner E. Cone and rod function in cone degenerations. Vis Res 1997;37:2303–14. 9578911 10.1016/s0042-6989(97)00025-4

[13] Day AC, Donachie PHJ, Sparrow JM, Johnston RL. The Royal College of Ophthalmologists’ National Ophthalmology Database study of cataract surgery: Report 1, visual outcomes and complications. Eye 2015;29:552–60. 25679413 10.1038/eye.2015.3 PMC4816350

[14] Vandenbroucke T, Buyl R, De Zaeytijd J, Bauwens M, Uvijls A, De Baere E, et al. Colour vision in stargardt disease. Ophthalmic Res. 2015;54:181–94. 26492201 10.1159/000438906

[15] Bonini Filho MA, Witkin AJ. Outer retinal layers as predictors of vision loss. Review of Ophthalmology. 2015. https://www.reviewofophthalmology.com/article/outer-retinal-layers-aspredictors-of-vision-loss

[16] Lima LH, Zett C, Kniggendorf V, Marianelli B, de Carvalho RAP, Farah ME, et al. Progressive expansion of the hyperautofluorescent ring in cone-rod dystrophy patients. Ophthalmic Genet. 2018;39:492–9. 29671671 10.1080/13816810.2018.1461911

[17] Oh JK, Ryu J, de Carvalho JRL, Levi SR, Lee W, Tsamis E, et al. Optical gap biomarker in cone-dominant retinal dystrophy. Am J Ophthalmol. 2020;218:40–53. 32445700 10.1016/j.ajo.2020.05.016 PMC8291221

[18] Cho SC, Woo SJ, Park KH, Hwang J. Morphologic characteristics of the outer retina in cone dystrophy on spectral-domain optical coherence tomography. Korean J Ophthalmol. 2013;27:19–27. 23372375 10.3341/kjo.2013.27.1.19 PMC3550307

[19] Thiadens AA, Phan TM, Zekveld-Vroon RC, Leroy BP, van den Born LI, Hoyng CB, et al. Clinical course, genetic aetiology, and visual outcome in cone and cone–rod dystrophy. Ophthalmology. 2012;119:819–26. 22264887 10.1016/j.ophtha.2011.10.011

[20] Armstrong RA. When to use the Bonferroni correction. Ophthalmic Physiol Opt. 2014;34:502–8. 10.1111/opo.12131. 10.1111/opo.12131 24697967

[21] Mustafi D, Engel AH, Palczewski K. Structure of cone photoreceptors. Prog Retin Eye Res. 2009;28:289–302. 19501669 10.1016/j.preteyeres.2009.05.003 PMC2740621

[22] Foote KG, Loumou P, Griffin S, Qin J, Ratnam K, Porco TC, et al. Relationship between foveal cone structure and visual acuity measured with adaptive optics scanning laser ophthalmoscopy in retinal degeneration. Invest Ophthalmol Vis Sci. 2018;59:3385–93. 30025078 10.1167/iovs.17-23708 PMC6038831

[23] Dain SJ. Clinical colour vision tests. Clin Exp Optom. 2004;87:276–93. 15312031 10.1111/j.1444-0938.2004.tb05057.x

[24] French A, Rose K, Thompson K, Cornell E. The evolution of colour vision testing. Aust Orthopt J. 2008;40:7–15.

