# Supplementary material for: Hardy-Rand-Rittler colour vision testing in cone and cone-rod dystrophies: correlation with structural and functional outcome measures
Source: Eye (Lond). 2025 Jan 16;39(3):527–32. doi: 10.1038/s41433-024-03584-2 (PMC11794465; doi:10.1038/s41433-024-03584-2)
Supplement: Supplementary file 1 — Supplementary Figure 1 - Hardy-Rand-Rittler Scoring Sheet [file 41433_2024_3584_MOESM1_ESM.docx]

Supplementary Figure 1 – Hardy-Rand-Rittler Scoring Sheet


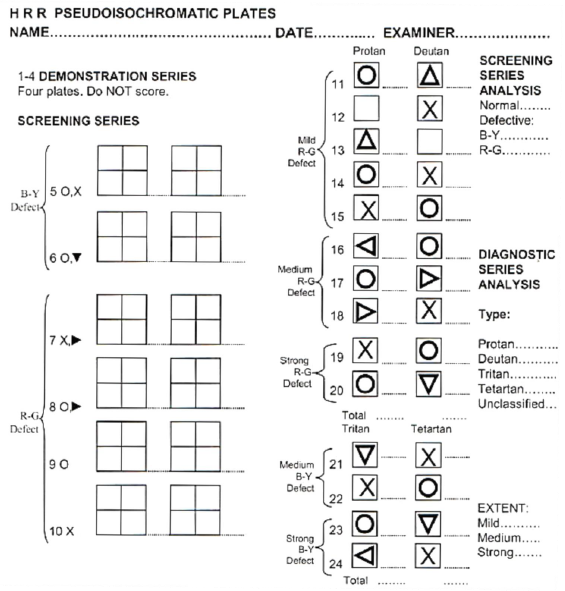


*B-Y* Blue-Yellow; *R-G* Red-Green

Permission to reuse this image has been obtained from Good-Lite; the company who published the above scoring sheet.
